# Association between kidney stones and life's essential 8: a population-based study

**DOI:** 10.1007/s00345-024-04994-3

**Published:** 2024-04-30

**Authors:** Yuan-Zhuo Du, Biao Guo, Hong-Ji Hu, Qian-Xi Dong, Yi-He Li, Ji Zhang, Fu-Chun Li, Ju Guo

**Affiliations:** 1 Department of Urology, The First Affiliated Hospital of Nanchang University, Nanchang, Jiangxi Province China; 2 Jiangxi Institute of Urology, Nanchang, Jiangxi Province China

**Keywords:** Kidney stones, Life's essential 8, NHANES, Cardiovascular health (CVH)

## Abstract

**Background:**

Kidney stones exhibit a robust correlation with cardiovascular disease (CVD). The objective of this research is to investigate the correlation between kidney stones and Life's Essential 8 (LE8), a newly updated assessment of cardiovascular health (CVH), among adults in the United States.

**Methods:**

In this study, which analyzed data from the 2007–2018 National Health and Nutrition Examination Survey, we employed LE8 scores (ranging from 0 to 100) as the independent variable, classifying them into low, moderate, and high CVH categories. The research examined the relationship between LE8 scores and kidney stones by using multivariate logistic regression and restricted cubic spline models, with kidney stones as the dependent variable.

**Results:**

Out of the 14,117 participants in this research, the weighted mean LE8 score was 69.70 ± 0.27. After accounting for confounding factors, there was an inverse association between higher LE8 scores and the likelihood of developing kidney stones (OR of 0.81 per 10-point increase, with a 95% confidence interval of 0.77–0.85), demonstrating a non-linear dose–response pattern. Similar patterns were observed for health behaviors, health factor scores, and kidney stones. Stratified analyses demonstrated a stable negative correlation between LE8 scores and kidney stones across different subgroups.

**Conclusion:**

LE8 and its subscale scores exhibited a robust and inverse correlation with the occurrence of kidney stones. Encouraging adherence to optimal CVH levels has the potential to serve as an effective strategy in preventing and minimizing the occurrence of kidney stones.

**Supplementary Information:**

The online version contains supplementary material available at 10.1007/s00345-024-04994-3.

## Introduction

Globally, kidney stones, which are mineral deposits that form in the renal calyces and pelvis, represent a significant health challenge [1]. It's noteworthy that the prevalence of kidney stones is increasing worldwide. For instance, in the United States, the incidence doubled from 1964 to 1972, reaching 8.8% in the past decade [2, 3]. Recently, the prevalence has risen to 11% among individuals over the age of 20, with a higher incidence in men than in women [4–6]. Factors contributing to this condition include insufficient water intake, high salt and protein diets, and obesity [7–10]. Although past research has suggested a link between an increased risk of cardiovascular events and kidney stones [11], delving further into the intricate relationship between cardiovascular health and kidney stones is crucial.

The rationale for exploring the correlation between cardiovascular health and kidney stones stems from the potential interplay between systemic factors affecting both conditions. Previous research has suggested shared risk factors such as obesity, hypertension, and metabolic syndrome, which are known to contribute to both cardiovascular disease and kidney stone formation [12]. Additionally, studies have indicated that individuals with a history of kidney stones may be at an increased risk of developing cardiovascular complications, suggesting a possible underlying physiological connection [11, 13]. Therefore, understanding the association between cardiovascular health and kidney stones is paramount for elucidating the complex pathophysiological mechanisms underlying both conditions.

In 2010, the American Heart Association (AHA) presented Life's Simple 7 (LS7) as a measure to evaluate cardiovascular health (CVH) [14]. In order to improve overall public health, the AHA has recently revised the CVH evaluation instrument to Life's Essential 8 (LE8), making improvements from the initial LS7 version.LE8 incorporates sleep quality indicators and enhanced scoring algorithms [15]. Highlighting the importance of maintaining or improving CVH in relation to social factors influencing health and psychology, LE8 emphasizes its enhanced ability to detect variations among individuals [16]. While the connection between LE8 and extended CVD-free survival, overall lifespan, and enhanced quality of life has been established[15], no research has been conducted on its correlation with kidney stones.

This study aimed to investigate the correlation between LE8 and kidney stones in a nationally representative group of U.S. adults, utilizing the latest NHANES (National Health and Nutrition Examination Survey) data. The objective was to provide new insights into the potential link between cardiovascular health and kidney stones, building on the established association between the two [11, 13].

## Methods

### Study population

Data from the NHANES covering the years 2007 to 2018 were utilized in this cross-sectional study. The NHANES database undergoes biennial data collection. All the databases could be obtained from the NHANES website (https://wwwn.cdc.gov/nchs/nhanes/Default.aspx). Written informed consent was obtained from participants after the study was approved by the Ethics Review Committee of the National Center for Health Statistics. All procedures for this study were conducted in accordance with relevant guidelines and regulations (https://www.cdc.gov/nchs/data_access/restrictions.htm). Adhering to relevant guidelines and regulations, 59,842 subjects were initially screened. Exclusion criteria included age under 20 years (n = 25,072), missing LE8 data (n = 11,154), missing values regarding renal stones (n = 53), and the presence of covariates with missing data (n = 9,446). This resulted in a final sample of 14,117 subjects for analysis (Supplement Fig. 1).

**Fig.1:**
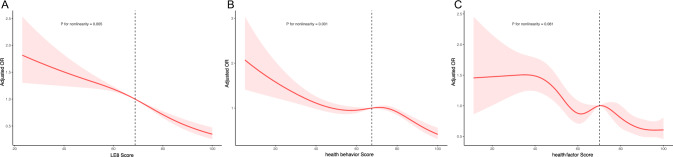
illustrates the correlation between Overall CVH (**A**), Health Behavior Score (**B**), Health Factors Score (**C**), and the occurrence of Kidney Stones. The ORs, represented by solid lines, were adjusted for sex, age, race, education levels, marital status, poverty ratio, alcohol intake and CVD, while their corresponding 95% confidence intervals (shaded areas) were also taken into account. The minimal threshold for a positive association is indicated by vertical dotted lines, which correspond to an estimated OR of 1

### Measurement of the LE8

LE8 comprises four health behaviors (diet, physical activity, nicotine exposure, and sleep duration) and four health factors (body mass index, blood lipids, blood glucose, and blood pressure). Dietary indicators were evaluated using the 2015 Healthy Eating Index 24 (HEI) through a 15-h dietary review of subjects [17]. Physical activity, nicotine exposure, sleep duration, and diabetes status were collected through the questionnaire. Data from the laboratory included blood lipids, blood glucose, and weight, while results from the mobile examination center (MEC) included blood pressure, height, and weight. Detailed algorithms for calculating the LE8 scores for each of the metrics to NHANES data have been previously published and show in Supplementary Table 1 [15, 16]. Each of the eight CVH metrics was scored using a new scoring method with a range of 0–100 points. The total LE8 score was then calculated as the unweighted mean of the eight metrics. LE8 scores of 80–100 were assigned to subjects with high CVH, 50–79 were assigned to those with moderate CVH, and 0–49 were assigned to individuals with low CVH [15].

### Measurement of kidney stones

A history of kidney stones is defined as "Have you ever had kidney stones?". The accuracy of self-reported kidney stone status has been validated in prior studies [18]. Participants were categorized as having a history of kidney stones if they responded affirmatively ("yes").

### Definition of covariates

As part of our study, a number of factors that have previously been identified as associated with LE8 or kidney stones were included as covariates. These factors included age strata (from 20 to 39 years, from 40 to 59 years, from 60 years up), sex (male, female), race (Mexican American, non-Hispanic black, non-Hispanic white, other Hispanic, other races), marital status (divorced/separated/widowed, married/living with a partner, never married), education levels (High school and below, Above high school), poverty ratio (less than 1.3, between 1.3 and 3.5, greater than 3.5), alcohol intake (categorized as "no" for participants with fewer than 12 drinks in the past 12 months and "yes" for those with at least 12 drinks in the past 12 months) [19], and cardiovascular disease(CVD). Cardiovascular disease was identified through self-report [20].

### Statistical analysis

Statistical analysis was performed using R (version 4.3.2). Data were analyzed according to NHANES data analysis guidelines, utilizing recommended survey weights. Statistical analyses were conducted using survey-weighted logistic regression for continuous variables (mean, standard errors [SE]), and survey-weighted chi-square tests for categorical variables (number of counts, percentages). A multivariate logistic regression analysis was used to determine whether kidney stones were associated with LE8 groups (categorized into three groups). Three different models were created: crude model without any modifications, model 1which accounted for age strata, sex, and race, and model 2 which additionally considered education levels, marital status, alcohol intake, creatinine, urinary creatinine, and CVD, building upon the adjustments made in model 1. To validate the correlation between kidney stones and LE8 scores, a Restricted cubic spline (RCS) was utilized, and stratified analyses were conducted for different subgroups at baseline. We calculated the interaction between stratification factors and LE8 scores using a multiplicative interaction test. A two-sided P value < 0.05 considered statistically significant.

## Results

### Baseline characteristics

In Table 1, the baseline characteristics of the weighted demographics are shown for the low, medium, and high CVH subgroups. Out of the total 14,117 participants, 50.82% were male. The average age was 46.22 ± 0.30 years. The average LE8 score was 60.70 with a standard deviation of 0.27. The majority identified as non-Hispanic white (72.44%), with 1,562 (8.68%), 9,419 (65.34%), and 3,136 (25.98%) in low, medium, and high CVH categories. Compared to the low CVH group, members of the high CVH group were younger and mostly female. They also had higher levels of education, lower rates of divorce/separation/widowhood, greater affluence, and lower alcohol intake. Furthermore, people belonging to the high CVH category had decreased blood and urine creatinine levels in the normal range compared to medium CVH and low CVH. Kidney stone risk decreased as CVH scores increased.

**Table 1:** Baseline Characteristics of the study population

Life’s Essential 8 score (LE8)	Total	Low (LE8 < 50)	Moderate (50 ≤ LE8 < 80)	High (LE ≥ 80)	P-value
Participant number	14,117	1562(8.68)	9419(65.34)	3136(25.98)	…
Age, y, mean (SE)	46.22(0.30)	51.99(0.44)	47.37(0.32)	41.41(0.50)	< 0.0001
Age strata, y, n (%)					< 0.0001
20–39	5282(37.67)	290(20.79)	3269(34.72)	1723(50.72)	
40–59	4929(39.34)	679(47.66)	3332(40.19)	918(34.43)	
≥ 60	3906(22.99)	593(31.56)	2818(25.09)	495(14.85)	
Sex, n (%)					< 0.0001
Male	7393(50.82)	834(51.59)	5225(54.42)	1334(41.52)	
Female	6724(49.18)	728(48.41)	4194(45.58)	1802(58.48)	
Race n (%)					< 0.0001
Mexican American	1906(7.27)	195(7.01)	1326(7.59)	385(6.54)	
Non-Hispanic Black	2738(9.18)	433(15.00)	1948(10.05)	357(5.05)	
Non-Hispanic White	6696(72.44)	687(68.06)	4416(71.68)	1593(75.83)	
Other Hispanic	1335(4.92)	149(4.87)	905(4.97)	281(4.81)	
Other Race	1442(6.19)	98(5.05)	824(5.71)	520(7.76)	
Marital status, n (%)					< 0.0001
Divorced/Separated/Widowed	2780(16.76)	482(26.32)	1947(18.36)	351(9.56)	
Married/Living with a partner	8549(64.78)	857(59.83)	5782(64.99)	1910(65.93)	
Never married	2788(18.45)	223(13.86)	1690(16.65)	875(24.51)	
Education levels, n (%)					< 0.0001
High school and below	5515(31.92)	872(51.39)	3970(35.72)	673(15.87)	
Above high school	8602(68.08)	690(48.61)	5449(64.28)	2463(84.13)	
Alcohol intake, n (%)					0.003
No	5252(36.85)	534(35.51)	3428(35.74)	1290(40.08)	
Yes	8865(63.15)	1028(64.49)	5991(64.26)	1846(59.92)	
Poverty ratio, n (%)					< 0.0001
< 1.30	3758(17.06)	614(28.30)	2539(17.56)	605(12.05)	
1.30–3.50	5215(33.73)	632(41.04)	3578(35.02)	1005(28.04)	
> 3.50	5144(49.21)	316(30.65)	3302(47.42)	1526(59.91)	
Urinary creatinine (mg/dl), mean (SE)	120.45(1.10)	131.76(2.54)	125.02(1.40)	105.17(1.69)	< 0.0001
Creatinine (mg/dl), mean (SE)	0.88(0.00)	0.91(0.01)	0.89(0.00)	0.84(0.00)	< 0.0001
LE8 score, mean (SE)	69.70(0.27)	42.52(0.23)	66.44(0.14)	86.97(0.14)	< 0.0001
HEI-2015 diet score	39.74(0.57)	19.79(0.84)	34.56(0.55)	59.43(0.76)	< 0.0001
Physical activity score	77.45(0.52)	32.63(1.80)	75.97(0.59)	96.14(0.30)	< 0.0001
Nicotine exposure score	70.32(0.65)	38.15(1.50)	66.27(0.73)	91.26(0.52)	< 0.0001
Sleep health score	84.59(0.33)	67.01(1.02)	83.56(0.34)	93.04(0.36)	< 0.0001
Body mass index score	61.29(0.50)	30.37(0.97)	55.86(0.48)	85.27(0.51)	< 0.0001
Blood lipids score	65.02(0.44)	42.01(0.94)	60.79(0.53)	83.34(0.58)	< 0.0001
Blood glucose score	87.63(0.28)	63.04(0.95)	86.85(0.33)	97.79(0.21)	< 0.0001
Blood pressure score	71.57(0.41)	47.11(0.97)	67.70(0.50)	89.48(0.49)	< 0.0001
CVD					< 0.0001
No	13,808(98.48)	1472(95.16)	9215(98.45)	3121(99.68)	
Yes	309(1.52)	90(4.84)	204(1.55)	15(0.32)	
Kidney stone					< 0.0001
No	12,832(90.51)	1339(85.89)	8509(89.54)	2984(94.49)	
Yes	1285(9.49)	223(14.11)	910(10.46)	152(5.51)	

LE8 life’s essential 8, HEI healthy eating index, CVD cardiovascular disease

### LE8 score and kidney stones

After correction for age, a significantly lower prevalence of kidney stones was observed in the high Overall CVH (6.08 ± 0.61%) and moderate Overall CVH (10.18 ± 0.41%) than the low Overall CVH (13.99 ± 1.17%) (Supplementary Fig. 2A). After multifactorial correction, the odds ratios (ORs) were 0.76 (95% CI 0.59–0.97) and 0.43 (95% CI 0.31–0.58) in the moderate LE8 and high LE8 groups compared with the low LE8 group. OR of 0.81 (95% CI 0.77–0.85) was associated with every 10-point increase in LE8 score in relation to kidney stones (Table 2). We determined a non-linear association between LE8 score and kidney stones (non-linear p = 0.005; Fig. 1). The lowest threshold for a beneficial association was 68.8 points (estimated OR = 1).

**Table 2:** Association of the Life’s Essential 8 scores with kidney stone

Exposure	Crude model		Model 1		Model 2	
	OR (95% CI)	P value	OR (95% CI)	P value	OR (95% CI)	P value
LE8 score						
Low (0–49)	1.00 (Reference)	…	1.00 (Reference)	…	1.00 (Reference)	…
Moderate (50–79)	0.71(0.56,0.90)	0.0100	0.73(0.57,0.94)	0.0100	0.76(0.59,0.97)	0.0300
High (80–100)	0.35(0.27,0.47)	< 0.0001	0.40(0.30,0.54)	< 0.0001	0.43(0.31,0.58)	< 0.0001
Per 10-point increase	0.78(0.75,0.82)	< 0.0001	0.80(0.76,0.84)	< 0.0001	0.81(0.77,0.85)	< 0.0001
Health behaviors score						
Low (0–49)	1.00 (Reference)	…	1.00 (Reference)	…	1.00 (Reference)	…
Moderate (50–79)	0.83(0.70,0.99)	0.04	0.80(0.68,0.96)	0.01	0.81(0.68,0.97)	0.02
High (80–100)	0.62(0.51,0.76)	< 0.0001	0.57(0.47,0.69)	< 0.0001	0.58(0.47,0.72)	< 0.0001
Per 10-point increase	0.91(0.88,0.95)	< 0.0001	0.90(0.87,0.93)	< 0.0001	0.90(0.87,0.94)	< 0.0001
Health factors score						
Low (0–49)	1.00 (Reference)	…	1.00 (Reference)	…	1.00 (Reference)	…
Moderate (50–79)	0.66(0.55,0.79)	< 0.0001	0.68(0.57,0.82)	< 0.001	0.70(0.58,0.84)	< 0.001
High (80–100)	0.37(0.29,0.46)	< 0.0001	0.45(0.36,0.57)	< 0.0001	0.48(0.38,0.61)	< 0.0001
Per 10-point increase	0.83(0.80,0.86)	< 0.0001	0.86(0.82,0.90)	< 0.0001	0.87(0.83,0.91)	< 0.0001

Crude model: unadjusted model

Model 1: Adjusted for age, sex, race

Model 2: Additionally adjusted for alcohol intake, education levels, marital status, poverty ratio, urinary creatinine, creatinine

*OR* odds ratio, *CI* confidence interval, *LE8* life’s essential 8

### Health behavior scores and kidney stones

After correction for age, it was found that people with high health behavior (7.42 ± 0.49%) tended to have fewer kidney stones than those with moderate (9.99 ± 0.42%) or low (11.76 ± 0.72%) health behavior (Supplementary Fig. 2B). In multivariate regression analyses, kidney stone ORs were 0.81 (95% CI 0.68–0.97) and 0.58 (95% CI 0.47–0.72) in the moderate health behavior and high health behavior groups, respectively, compared with the low health behavior group. OR of 0.90 (95% CI 0.87–0.94) was associated with every 10-point increase in Health behavior scores in relation to kidney stones (Table 2). There was a nonlinear association between health behavior scores and kidney stones (nonlinear p = 0.001; Fig. 1). The minimum threshold for a beneficial association was 67.5 points (estimated OR = 1).

### Health factors and kidney stones

After correction for age, Subjects with high health factors exhibited a significantly lower prevalence of kidney stones (7.17 ± 0.59%) compared to those with moderate (10.04 ± 0.46%) and low health factors (14.15 ± 0.97%) (Supplementary Fig. 2C). After multivariable adjustment, kidney stone ORs were 0.70 (95% CI 0.58–0.84) and 0.48 (95% CI 0.38–0.61) in the moderate and high health factor groups, respectively, compared with the low health factor group. OR of 0.87 (95% CI 0.83–0.91) was associated with every 10-point increase in health factors scores in relation to kidney stones (Table 2). However, there was no nonlinear association between health factor scores and kidney stones (P = 0.081; Fig. 1). The minimum threshold for a beneficial association was 70.0 points (estimated OR = 1).

### Subgroup analysis

Subgroup analyses support a consistent negative association between LE8 scores and kidney stones (Supplementary Fig. 3). The correlation remains consistent across different categories, such as sex, age strata, race, education levels, marital status, poverty ratio, alcohol intake, and CVD, regardless of these variables (p-values for all interactions > 0.05). These results suggest a consistently negative association across diverse demographics, indicating potential broader applicability in various population settings.

## Discussion

In this study, we conducted a detailed exploration of the relationship between LE8 scores and the prevalence of adult kidney stones, and found a significant negative correlation between the two. This finding reveals for the first time the potential of LE8 scores as a comprehensive cardiovascular health indicator for reducing the risk of kidney stones. As a comprehensive assessment of cardiovascular health (CVH), LE8 is more comprehensive than the previous LS7 score and has been shown to be superior in predicting stroke risk and arterial stiffness.[15, 16, 21]. This study first reveals the potential connection between LE8 scores and the risk of kidney stones, providing a new perspective for further exploration of the value of comprehensive health behaviors and factors in preventing kidney stones.

Our research complements existing literature, indicating the importance of multidimensional health assessment in reducing the risk of kidney stones. Previous studies have shown that factors such as diet, physical activity, smoking, and sleep quality are associated with the risk of kidney stones, and our study further emphasizes the necessity of comprehensive assessment of these behaviors. For example, adequate fluid intake, balanced dietary structure, [22], moderate physical activity [23], smoking cessation[24], and maintaining normal sleep time are considered effective measures to reduce the risk of kidney stones [25].

Furthermore, our research highlights the importance of optimizing health factors—BMI, lipids, blood sugar, and blood pressure—in reducing the risk of kidney stones. We found that the risk of kidney stone formation is higher in obese or overweight individuals, directly associated with an increase in the risk of kidney stones [12]. Similarly, abnormal elevation of lipid levels is also associated with increased risk of kidney stones [26]. An increase in blood sugar levels, even within the normal range, has been found to be associated with an increased risk of kidney stone formation [27]. Additionally, hyperuricosuria and hypercalciuria, along with related calcium oxalate and uric acid supersaturation, appear to be particularly important in hypertensive patients, further emphasizing the importance of cardiovascular health maintenance in preventing kidney stones [28].

Moreover, our research highlights the potential value of LE8 scores in promoting interdisciplinary cooperation [29], especially between urologists and cardiologists. By identifying lifestyle and health behavior factors associated with increased cardiovascular disease risk, LE8 scores can serve not only as a tool for predicting cardiovascular disease but also as a new strategy for preventing kidney stones [30]. This interdisciplinary screening and management approach will help achieve a comprehensive assessment and intervention for patient health status. Given the correlation between LE8 scores and the risk of kidney stones, we believe that incorporating this score into routine health checks can provide important clues for early identification and intervention. Furthermore, considering the widely recognized connection between kidney stones and cardiovascular disease [11], our findings further reinforce the importance of maintaining good cardiovascular health in preventing kidney stones. Although this study provides important insights, we also acknowledge its limitations as a cross-sectional study and its inability to establish causality. Therefore, future research should include larger prospective cohort studies to validate the relationship between LE8 scores and the risk of kidney stones and consider potential confounding factors. Additionally, future research should explore how to translate the application of LE8 scores from theoretical research to clinical practice and how to achieve personalized health management measures in different populations.

## Conclusions

In this cross-sectional study, we found that elevated LE8 scores, health behavior scores, and health factor scores were significantly and negatively associated with kidney stones. These results underscore the potential effectiveness of LE8 as a preventive measure against kidney stones, highlighting the importance of maintaining cardiovascular health to reduce the occurrence of kidney stones. Additional research is necessary to explore the cause-and-effect relationship and clarify the exact mechanisms linking LE8 and kidney stones in forthcoming studies.

## Supplementary Information

Below is the link to the electronic supplementary material.

Supplementary file1 (DOCX 181 KB)

## Data Availability

All data used in this study are available in the NHANES database, accessible at https://www.cdc.gov/nchs/nhanes/i
