# Supplementary material for: Association between kidney stones and life's essential 8: a population-based study
Source: World J Urol. 2024 Apr 30;42(1):274. doi: 10.1007/s00345-024-04994-3 (PMC11061036; doi:10.1007/s00345-024-04994-3)
Supplement: Supplementary file 1 — Supplementary file1 (DOCX 181 KB) [file 345_2024_4994_MOESM1_ESM.docx]

**Supplementary Material**

**Association between Kidney Stones and life's essential 8: a population-based study**

Yuan-Zhuo Du^1,2^, Biao Guo^1,2^, Hong-Ji Hu^1,2^, Qian-Xi Dong^1,2^, Yi-He Li^1,2^, Ji Zhang^1,2^, Fu-Chun Li^1,2^, Ju Guo^1,2*^

^1^The First Affiliated Hospital of Nanchang University's Urology Department is located in Nanchang, Jiangxi Province, China with the postal code 330000.

^2^Jiangxi Institute of Urology, Nanchang 330000, Jiangxi Province, China.

* Correspondence: Ju Guo^1,2*^

^1^The First Affiliated Hospital of Nanchang University's Urology Department is located in Nanchang, Jiangxi Province, China with the postal code 330000

^2^Jiangxi Institute of Urology, Nanchang 330000, Jiangxi Province, China.

Ju Guo^1,2*^ Email: ndyfy02371@ncu.edu.cn

******Supplementary Fig.1: The participant flow chart**

**Supplementary** **Table 1: Definition and scoring approach for the American Heart Association’s Life’s Essential 8 score.**

| Domain | CVH Metric | Measurement | Quantification and Scoring of CVH Metric |
| --- | --- | --- | --- |
| Health Behaviors | Diet | Healthy Eating Index-2015 diet score percentile | Quantiles of DASH-style diet adherence  **Scoring (Population):**  Points Quantile  100 ≥95^th^ percentile (top/ideal diet)  80 75^th^ – 94^th^ percentile  50 50^th^ – 74^th^ percentile  25 25^th^ – 49^th^ percentile  0 1^st^ – 24^th^ percentile (bottom/least ideal quartile) |
|  | Physical activity | Self-reported minutes of moderate or vigorous physical activity per week | **Metric:** Minutes of moderate (or greater) intensity activity per week  **Scoring:**  Points Minutes  100 ≥150  90 120 – 149  80 90 – 119  60 60 – 89  40 30 – 59  20 1 – 29  0 0 |
|  | Nicotine exposure | Self-reported use of cigarettes or inhaled nicotine- delivery system | **Metric:** Combustible tobacco use and/or inhaled NDS use; or secondhand smoke exposure  **Scoring:**  Points Status  100 Never smoker  75 Former smoker, quit ≥5 yrs  50 Former smoker, quit 1 - <5 yrs  25 Former smoker, quit <1 year, or currently using inhaled NDS  0 Current smoker  Subtract 20 points (unless score is 0) for living with active indoor smoker in home |
|  | Sleep health | Self-reported average hours of sleep per night | **Metric:** Average hours of sleep per night  **Scoring:**  Points Level  100 7 – <9  90 9 – <10  70 6 – <7  40 5 – <6 or ≥10  20 4 – <5  0 <4 |
| Health Factors | Body mass index | Body weight (kg) divided by height squared (m^2^) | **Metric:** Body mass index (kg/m^2^)  **Scoring:** Points Level 100 <25  70 25.0 – 29.9  30 30.0 – 34.9  15 35.0 – 39.9  0 ≥40.0 |
|  | Blood lipids | Plasma total and HDL-cholesterol with calculation of non-HDL-cholesterol | **Metric:** Non-HDL-cholesterol (mg/dL)  **Scoring:**  Points Level  100 <130  60 130 – 159  40 160 – 189  20 190 – 219  0 ≥220  If drug-treated level, subtract 20 points |
|  | Blood glucose | Fasting blood glucose or casual hemoglobin A1c | **Metric:** Fasting blood glucose (mg/dL) or Hemoglobin A1c (%)  **Scoring:**  Points Level  100 No history of diabetes and FBG <100 (or HbA1c < 5.7)  60 No diabetes and FBG 100 – 125 (or HbA1c 5.7-6.4) (Pre-diabetes)  40 Diabetes with HbA1c <7.0  30 Diabetes with HbA1c 7.0 – 7.9  20 Diabetes with HbA1c 8.0 – 8.9  10 Diabetes with Hb A1c 9.0 – 9.9  0 Diabetes with HbA1c ≥10.0 |
|  | Blood pressure | Appropriately measured systolic and diastolic blood pressure | **Metric:** Systolic and diastolic blood pressure (mm Hg)  **Scoring:**  Points Level  100 <120/<80 (Optimal)  75 120-129/<80 (Elevated)  50 130-139 or 80-89 (Stage I HTN)  25 140-159 or 90-99  0 ≥160 or ≥100  Subtract 20 points if treated level |

Reference

1. Lloyd-Jones DM, Allen NB, Anderson CAM, et al. Life's Essential 8: Updating and Enhancing the American Heart Association's Construct of Cardiovascular Health: A Presidential Advisory From the American Heart Association. Circulation. Aug 2 2022;146(5):e18-e43.
2. Lloyd-Jones DM, Ning H, Labarthe D, et al. Status of Cardiovascular Health in US Adults and Children Using the American Heart Association's New "Life's Essential 8" Metrics: Prevalence Estimates From the National Health and Nutrition Examination Survey (NHANES), 2013 Through 2018. Circulation. Sep 13 2022;146(11):822-835.

**Supplement Fig.2: Age-adjusted prevalence of kidney stones at different Life’s Essential 8 for score levels**

Numbers at the top of the bar indicating weighted percentages and horizontal bars on the bar indicating 95% confidence levels.

**Supplementary Fig.3: Presents a stratified analysis of kidney stones and the Life's Essential 8 score**

OR is calculated for every 10 points added to the LE8 score. The analysis accounted for factors such as sex, age, race, education levers, marital status, poverty ratio, alcohol intake and CVD. OR odds ratio, CI confidence interval.
